# Engineering the Cellular Microenvironment of Post-infarct Myocardium on a Chip

**DOI:** 10.3389/fcvm.2021.709871

**Published:** 2021-07-14

**Authors:** Natalie N. Khalil, Megan L. McCain

**Affiliations:** ^1^Laboratory for Living Systems Engineering, Department of Biomedical Engineering, USC Viterbi School of Engineering, University of Southern California, Los Angeles, CA, United States; ^2^Department of Stem Cell Biology and Regenerative Medicine, Keck School of Medicine of USC, University of Southern California, Los Angeles, CA, United States

**Keywords:** tissue engineering, myocardial infarction, organ on a chip, hypoxia, stiffness, strain, cardiac myocytes, cardiac fibroblasts

## Abstract

Myocardial infarctions are one of the most common forms of cardiac injury and death worldwide. Infarctions cause immediate necrosis in a localized region of the myocardium, which is followed by a repair process with inflammatory, proliferative, and maturation phases. This repair process culminates in the formation of scar tissue, which often leads to heart failure in the months or years after the initial injury. In each reparative phase, the infarct microenvironment is characterized by distinct biochemical, physical, and mechanical features, such as inflammatory cytokine production, localized hypoxia, and tissue stiffening, which likely each contribute to physiological and pathological tissue remodeling by mechanisms that are incompletely understood. Traditionally, simplified two-dimensional cell culture systems or animal models have been implemented to elucidate basic pathophysiological mechanisms or predict drug responses following myocardial infarction. However, these conventional approaches offer limited spatiotemporal control over relevant features of the post-infarct cellular microenvironment. To address these gaps, Organ on a Chip models of post-infarct myocardium have recently emerged as new paradigms for dissecting the highly complex, heterogeneous, and dynamic post-infarct microenvironment. In this review, we describe recent Organ on a Chip models of post-infarct myocardium, including their limitations and future opportunities in disease modeling and drug screening.

## Introduction

Cardiovascular disease is the leading cause of death worldwide, responsible for over 17.9 million deaths annually (1). One of the most common causes of cardiovascular disease is coronary artery disease, in which plaque buildup in the coronary arteries deprives downstream myocardium of vital, oxygenated blood. Complete occlusion of the coronary arteries can ultimately lead to myocardial infarction, commonly referred to as a heart attack. Myocardial infarction is among the top five most expensive conditions treated by US hospitals annually and is a common cause of heart failure (2–4).

When a patient presents with a myocardial infarction, percutaneous coronary intervention is often performed, which is a catheterization procedure to restore flow to the interrupted artery and minimize the initial insult (5, 6). Rapid identification of myocardial infarction is essential for timely reperfusion of the occluded coronary artery, which can, in some cases, lead to reperfusion injury that further increases the size of the initial insult (7). After revascularization procedures, treatment is predominantly focused on pharmaceutical interventions to reduce adverse cardiovascular events, including stroke, recurrent infarctions, and death (8). Common treatments include renin-angiotensin-aldosterone system (RAAS) inhibitors, such as angiotensin-converting enzyme inhibitors, which decrease the load on the heart by lowering blood pressure (9) and may also reduce fibrotic remodeling (10). Another therapeutic target is beta adrenergic receptors, which are activated by epinephrine or norepinephrine to stimulate heart rate, strength of contraction, and cardiac output. Similar to RAAS inhibitors, beta blockers, such as bisoprolol, carvedilol, and metoprolol, can reduce blood pressure following myocardial infarction (8, 10). Thus, existing pharmacological interventions are primarily focused on reducing the load on the damaged heart instead of attempting to repair the initial injury or mitigate the ensuing fibrotic remodeling process.

One challenge facing the development of new therapies for post-infarct myocardium is that conventional preclinical models are limited primarily to non-human animal models or static, uniform monolayers of cultured cells that poorly replicate the clinical setting, especially the complex, dynamic remodeling that occurs post-infarction (3). Thus, developing the next generation of treatments for myocardial infarction can be accelerated by new preclinical model systems that more closely replicate native pathophysiology across multiple spatial scales, including the post-infarct cellular microenvironment.

Organs on Chips are engineered *in vitro* systems that mimic the fundamental structural and functional units of native tissues and have been shown to mimic responses to drugs at clinical doses in various tissue models with higher fidelity than conventional *in vitro* methods (11). Organs on Chips can also provide enhanced spatiotemporal control over multiple distinct features of the cellular microenvironment, which is especially relevant to modeling post-infarct myocardium. Thus, Organ on Chip approaches have vast potential to provide new mechanistic insights into post-infarct remodeling and to inform future pharmacological interventions.

In this review, we will first describe the cellular and molecular remodeling that occurs after myocardial infarction in humans, which will provide a framework for the microenvironmental features that are important to model on a chip. Next, we will describe existing strategies for engineering aspects of the cellular microenvironment of post-infarct myocardium on a chip, which have until now focused on hypoxia, fibrosis, and strain in both 2-dimensional (2-D) and 3-dimensional (3-D) tissue constructs. Lastly, we will describe considerations for future work to promote clinical mimicry and translation of Organ on a Chip models of myocardial infarction.

## Ventricular Remodeling Post-myocardial Infarction

The initial coronary occlusion of a myocardial infarction deprives downstream myocardium of vital oxygen and nutrients, causing cardiac myocyte necrosis within hours (10, 12, 13). Due to tissue necrosis, the infarct zone is vulnerable to deformation and thinning, which can lead to infarct expansion (14, 15). Cardiac myocytes in the surviving infarct border zone myocardium also begin to rapidly remodel. For example, the normal distribution of intercalated disc protein complexes (gap junctions, desmosomes, and adherens junctions) is lost as early as 6 h post-infarction. Because intercalated disks are necessary for the heart to function as an electromechanical syncytium, changes in their expression and localization are thought to contribute to arrhythmogenesis after infarction (16).

As a result of infarct expansion, diastolic and systolic wall stresses increase (17, 18). To normalize the increased load, the myocardium undergoes cardiac hypertrophy in the weeks and months after an infarction by increasing muscle mass and wall thickness (19). These changes are reflected in the morphology of individual cardiac myocytes, which can increase in both length and diameter. Due to the spatial arrangement of cardiac myocytes in the ventricles, increased diameter and lengthening of individual cells ultimately results in changes in chamber geometry (6, 20, 21), which contributes to ventricular enlargement. Increasing chamber volume through ventricular enlargement may be compensatory in order to maintain stroke volume after the initial loss of contractility (14, 22) but is ultimately associated with increased likelihood of mortality (14, 23).

**Figure 1:**
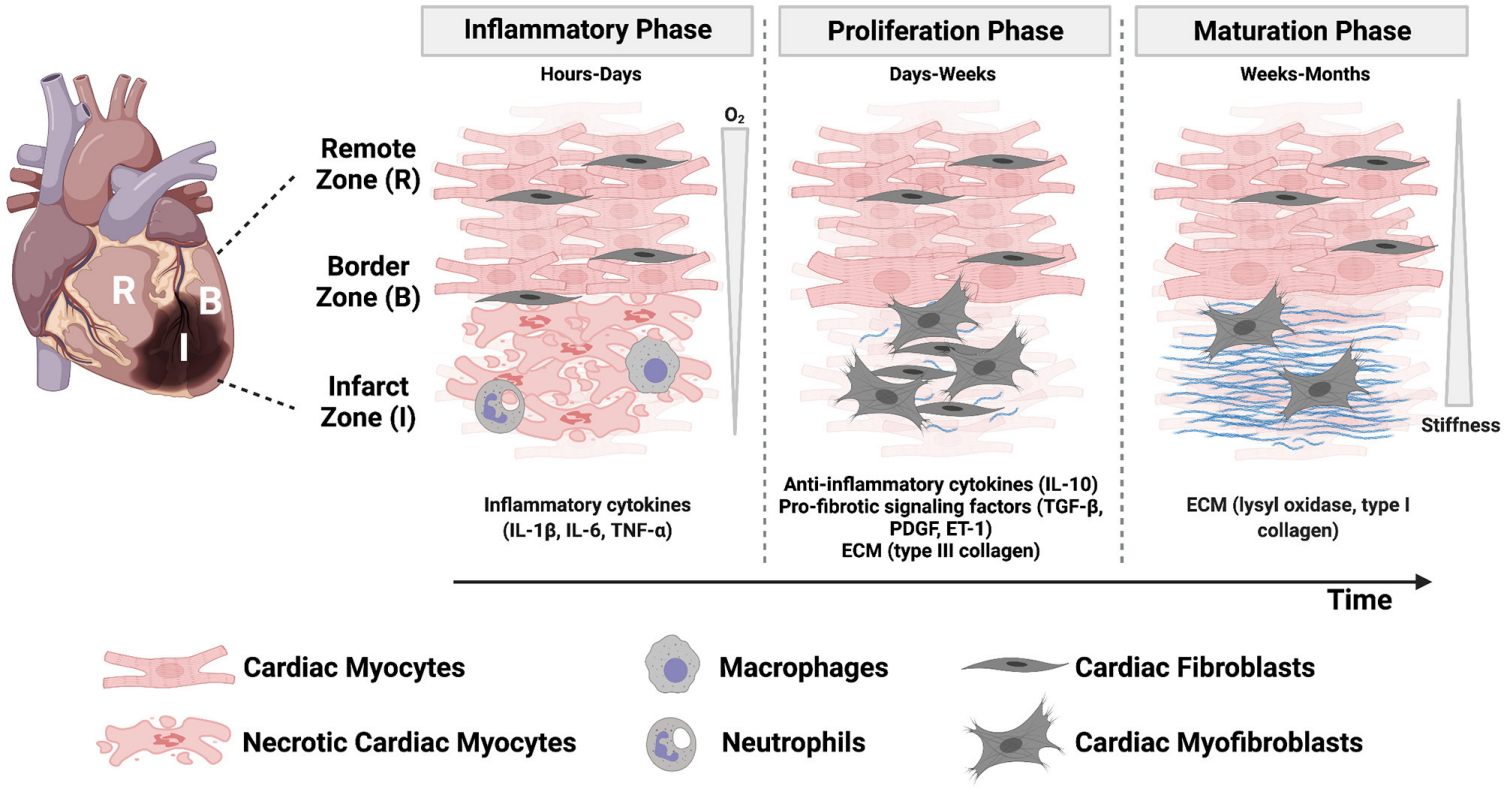
The post-infarct microenvironment is characterized by dynamic physical, mechanical, and biochemical cues that orchestrate the three phases of healing. Regional and temporal changes of oxygen concentration, stiffness, inflammatory cytokines, cell, and matrix composition characterize the myocardium as injured tissue is replaced with a scar. Created with BioRender.com.

Tissue necrosis and infarct expansion can also increase the likelihood of myocardial rupture (14, 15). Because mammalian cardiac myocytes have limited regenerative capacity, maintaining tissue integrity is dependent on the formation of a scar. Scar formation occurs through three overlapping phases: the inflammatory phase, proliferative phase, and maturation phase (Figure 1). The inflammatory phase occurs in the first few days following a myocardial infarction and is initiated when necrotic cardiac myocytes release their intracellular contents, which activate pro-inflammatory signaling pathways in innate immune cells (24). Next, inflammatory chemokine and cytokine gradients [comprised of tumor necrosis factor-α (TNF-α), interleukin (IL)-1β, and IL-6] promote leukocyte migration into the infarct zone to clear dead cell debris and damaged extracellular matrix. After 1 week, tissue inhibitors of metalloproteinases are upregulated to conclude this degradative phase (24). Ultimately, as neutrophils undergo apoptosis, macrophages are directed toward a resolving phenotype and begin secreting anti-inflammatory signals [such as transforming growth factor-β (TGF-β) and IL-10] that repress the inflammatory response and drive cardiac fibroblast activation in the subsequent proliferative phase (10).

The proliferative phase occurs over the next few weeks and is characterized by cardiac fibroblast proliferation, migration into the site of injury, and differentiation into an activated myofibroblast phenotype. Cardiac fibroblasts are the most abundant non-myocyte cell population in the mammalian myocardium (24) and, after expansion in the proliferative phase in combination with cardiac myocyte necrosis, they become the most abundant cell type in the infarcted region (10). Myofibroblasts play an important role in depositing collagen, fibronectin, and other matrix proteins to maintain tissue integrity and prevent myocardial rupture. They also express α-smooth muscle actin (α-SMA) and non-muscle myosin, which provide them with the ability to generate force to migrate and facilitate wound contracture (10).

The final phase is scar maturation, which occurs on a timescale of weeks to months. In this phase, myofibroblasts initiate collagen turnover and begin secreting type I collagen, which has the tensile strength of steel (12), in place of type III collagen. The depletion of growth factors necessary for myofibroblast survival ultimately leads to myofibroblast apoptosis (10, 25). Further enzymatic cross-linking of collagen occurs through lysyl oxidase, which progressively increases the tensile strength of the myocardium for months after myocardial infarction (10). While myofibroblasts play a vital role in maintaining structural integrity after the initial loss of tissue, they may also be driven by many cellular and molecular events toward a pathological fibrotic response. Myofibroblast persistence in the myocardium can occur for months, or even years, after injury and is a common feature of heart failure (12). Fibrotic tissue, which was historically considered an inert tissue, is now known to secrete factors (such as angiotensin II and TGF-β) that can traverse the interstitial space and promote fibrosis in non-infarcted regions (12, 26).

In summary, post-infarct myocardium is characterized by multiple biochemical and biomechanical properties changing in both space and time, which are correlated to complex remodeling of several cell types and the extracellular matrix in parallel (Figure 1). Some or all of these remodeling processes ultimately impact cardiac function and patient outcomes. However, the relationships between these biochemical, biomechanical, cellular, and molecular factors are incompletely understood, hindering the discovery of new therapies to mitigate the effects of the initial injury. Thus, there is a great need for controlled experimental models of post-infarct myocardium that account for remodeling of the cellular microenvironment to uncover mechanisms of pathophysiology.

## *In vitro* Models of Myocardial Ischemia and Hypoxia

Due to the high metabolic activity of cardiac myocytes, the myocardium is a highly vascularized tissue, with capillaries separated by approximately 20 μm (27). This translates to about one blood vessel between every two cardiac myocytes (28). As a result, hypoxia is one of the most injurious effects of a myocardial infarction. Conventionally, hypoxia has been induced by culturing cells in environments with uniformly low oxygen. However, phosphorescent oxygen probes have demonstrated that a spatial gradient ranging from 0 to 10% oxygen bridges injured tissue with neighboring viable tissue in post-infarct myocardium (29, 30). In addition, oxygen concentrations change over time as the infarct zone is re-oxygenated during reperfusion. Thus, *in vitro* systems that can modulate oxygen concentrations in space or time have more recently been developed to mimic the hypoxic landscape of post-infarct myocardium. In this section, we will describe conventional hypoxia models that replicate uniform hypoxia as well as engineered systems that offer spatial or temporal control over oxygen tension.

### Conventional Hypoxia Models

To recapitulate myocardial hypoxia *in vitro* (3), one of the most common approaches is to place cardiac cells in incubators or hypoxia chambers and replace oxygen with nitrogen. This enables the stable, long-term induction of hypoxia, with tunable control over global oxygen levels by selecting a gas composition of choice. To enable cell handling, hypoxia workstations have also been developed that contain gloveboxes to allow for the manipulation of cells in a hypoxic enclosure. However, these approaches for physical induction of hypoxia require access to specialized equipment, such as incubators with oxygen regulation, and are limited to uniform gas concentrations.

Hypoxia can also be simulated in cells cultured in ambient oxygen by adding hypoxia mimetic agents, such as cobalt chloride, to cell media. Hypoxia mimetic agents often work by stabilizing hypoxia inducible factors (HIF), a family of transcription factors that facilitates the cellular response to hypoxia by upregulating genes associated with survival in low oxygen. In normoxia, HIF is constantly degraded through hydroxylation of the HIF-α subunit by the enzyme prolyl hydroxylase, which marks it for ubiquitination by Von Hippel Lindau protein and subsequent degradation. Cobalt chloride chemically stabilizes HIF in normoxia by replacing Fe^2+^ with Co^2+^ in the prolyl hydroxylase active site, thereby inhibiting its hydroxylation of HIF-α and the ensuing degradation pathway. Other successful hypoxia mimetics include dimethyloxalylglycine and deferoxamine, which similarly work by inhibiting prolyl hydroxylase activity (31). Hypoxia mimetics enable easy access to cells during cell culture, are inexpensive, and can quickly simulate hypoxic conditions *in vitro*. However, they can have adverse effects on other signaling pathways that are not affected by low oxygen (31, 32), may be cytotoxic (33), and likely do not capture all the effects of true hypoxia.

Conventional *in vitro* models have shown that hypoxia results in structural and functional changes that may contribute to the development of arrhythmias. Specifically, the gap junction protein connexin 43 (Cx43) is known to be affected, which plays a critical role in conducting electrical impulses in the myocardium. Hypoxia has been shown to drive electrical uncoupling *in vitro*, with effects including decreased Cx43 signal at gap junctions (34, 35), increased Cx43 internalization (35) and dephosphorylation (36), and decreased conduction velocity (34). Hypoxia is also associated with inactivation of sodium potassium pumps (Na,K ATPase), which regulate cardiac action potentials (37, 38). Hypoxic cardiac myocytes also upregulate fetal, T type calcium channels, which are absent in healthy, adult myocardium, in a mechanism dependent on HIF-1α (39). Finally, hypoxia has also been shown to promote apoptosis in cardiac myocytes (40–43). These remodeling processes are possibly related to the increased incidence of arrhythmias observed in post-infarct myocardium.

Studies have also shown that cardiac fibroblasts and other non-myocyte cell populations are generally more resistant to hypoxia (40–42). In response to hypoxic stimuli *in vitro*, cardiac fibroblasts undergo differentiation into the activated, myofibroblast phenotype, which is signified by increased α-SMA expression (44–46), collagen type I expression (44–47), and migration capacity (46). However, there have been differing reports of cardiac fibroblast proliferation in response to hypoxia (44–46), which may result from differences in basal levels of fibroblast differentiation, or from differences in cell source, oxygen concentration, and hypoxia duration. In response to prolonged hypoxia exposure *in vitro*, cardiac fibroblasts ultimately undergo apoptosis (48–50).

Hypoxia also changes the secretion of paracrine factors that are involved in many processes of infarct healing, such as angiogenesis, fibroblast differentiation, and remodeling of the extracellular matrix. Hypoxic cardiac myocytes upregulate vascular endothelial growth factor (VEGF) (51, 52), insulin-like growth factor 2 (52), inflammatory cytokines (TNF-α, IL-1β, IL-6), and TGF-β (53). As TGF-β is known to promote fibroblast differentiation into myofibroblasts, medium conditioned by hypoxic myocytes has been reported to drive cardiac fibroblast migration (54) and facilitate faster wound closure in cultured skin fibroblasts (53). Similar to cardiac myocytes, hypoxic cardiac fibroblasts upregulate TGF-β1 and also its receptor, TGFβ-R1, which can play a role in autocrine signaling pathways to promote fibroblast differentiation (46, 48). Hypoxic fibroblasts also exhibit increased secretion of inflammatory cytokines [TNF-α (55–57) and IL-6 (57)], matrix metalloproteinases [MMP-2 and MMP-9 (56)] and VEGF (56), and conditioned medium from hypoxic fibroblasts has been shown to reduce cardiac myocyte viability (55, 58). Thus, hypoxia likely alters cellular cross-talk between distinct cardiac cell types in post-infarct myocardium.

### Engineered Models With Spatial Oxygen Gradients

Although conventional hypoxia systems have revealed valuable insights into oxygen-dependent remodeling of cardiac cell types, they do not replicate the spatial or temporal changes in oxygen that are characteristic of post-infarct myocardium. To mimic spatial oxygen gradients, more complex *in vitro* systems have been engineered. In one example, a microfluidic device was fabricated with a central channel designated for cell culture embedded between two lateral media channels. By flowing media containing a chemical hypoxia mimetic through one channel and standard media through the other, a chemical hypoxia gradient was established in the central cell-containing channel. Cardiac myoblasts near the hypoxic end of the gradient exhibited altered morphology, including reduced cell area and actin disintegration, which was accompanied by mitochondrial dysfunction and decreased cell viability (59). In addition to chemical methods (60–63), microfluidic devices have also been developed to generate physical oxygen gradients by culturing cells on a gas-permeable membrane above microchannels for gas flow (64–66). However, these methods have not been extensively applied to cardiac cell types.

Ischemic gradients have also been developed by stacking thin layers of hydrogels that are mechanically supported by a paper scaffold, a technique termed “cells-in-gels-in-paper.” To control oxygen and nutrient diffusion into the stack, one end of the construct was placed in a base that is impermeable to gases and liquids. Nutrients become depleted as they diffuse into the stack, which creates an ischemic environment in the lower layers. Cardiac myocytes in the ischemic, lower layers exhibited reduced viability and circular morphology when compared with upper layers. Cell-tracking demonstrated that cardiac fibroblasts embedded in upper layers migrate toward ischemic cardiac myocytes. Fibroblast migration increased when myocytes were exposed to higher levels of ischemia (generated through taller stacks) and was reduced in the absence of cardiac myocytes or with the pharmacological inhibition of TGF-β (27). Other methods have been developed that similarly modify hydrogels to generate oxygen gradients (67) by using oxygen-consuming enzymes during hydrogel cross-linking (68, 69), embedding a hydrogel between gas flow channels (70), or linearly increasing cell density and thus oxygen consumption rates (71), but these methods have not yet been implemented to model post-infarct myocardium.

**Figure 2:**
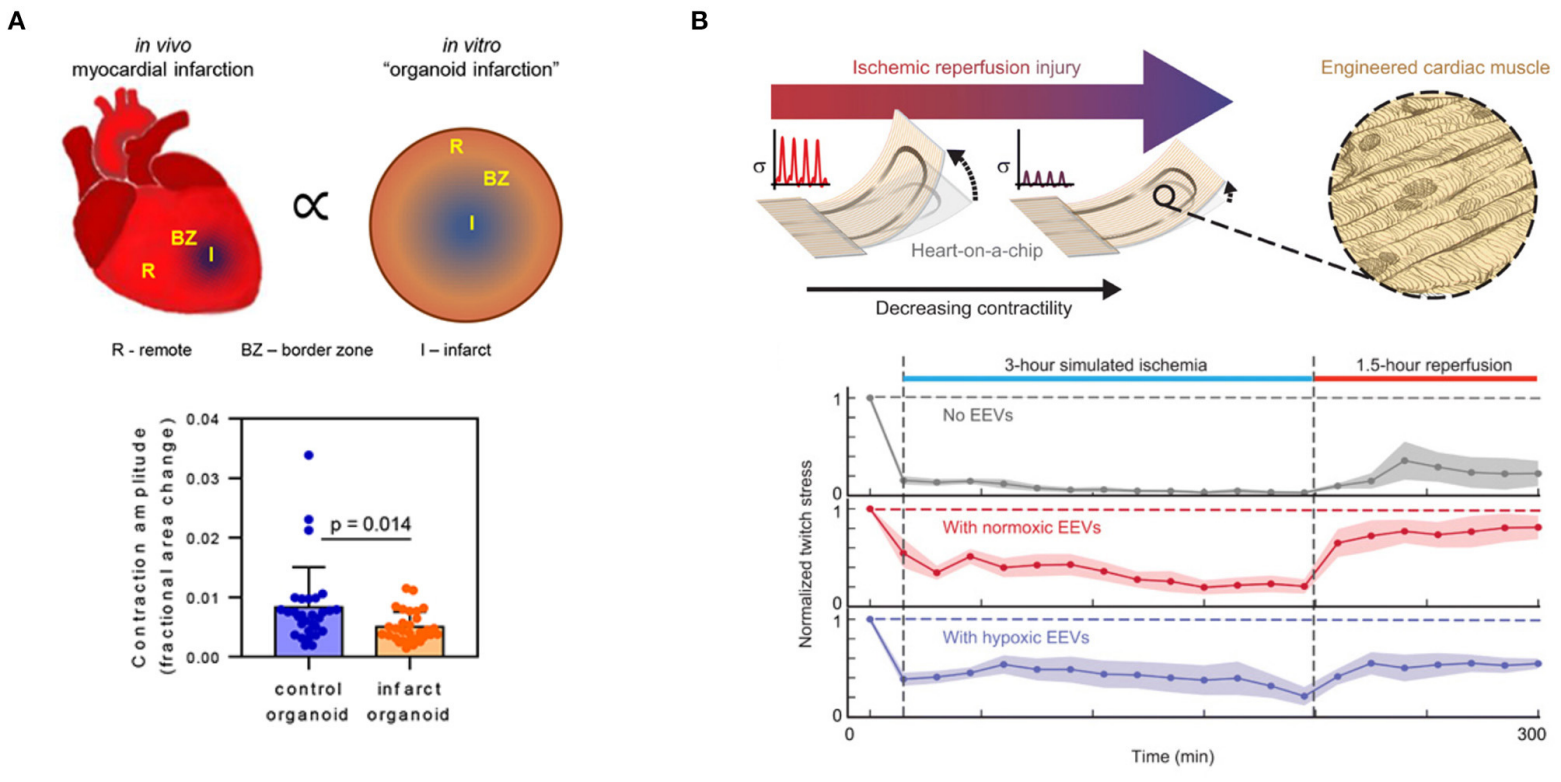
Engineered *in vitro* hypoxia models that modulate oxygen in **(A)** space and **(B)** time. **(A)** Infarct spheroids generate intrinsic spatial oxygen gradients to model the infarct, border, and remote zones after myocardial infarction. Infarct spheroids exhibit higher levels of myofibroblast marker expression, exhibit asynchronous contraction of myocytes, and have lower contraction amplitudes than control spheroids. Reprinted by permission from Springer Nature: Nat. Biomed. Eng. (78). **(B)** Muscular thin films with integrated strain gauges can measure changes in contractility in cardiac myocytes pre-treated with endothelial extracellular vesicles in a simulated ischemia-reperfusion injury. Endothelial extracellular vesicles enhanced the contractility of engineered cardiac tissues during both ischemia and reperfusion. From Yadid et al. (79). Reprinted with permission from AAAS.

Lastly, cardiac spheroids have also been implemented to mimic hypoxia gradients. Cardiac spheroids are 3-D aggregates of cardiac cells that recapitulate select aspects of native tissue structure and function (72–76). Because the diffusion limit of oxygen in tissues is around 100–200 microns (77), cardiac spheroids intrinsically generate oxygen gradients, for which oxygen tension is highest at the surface and decreases toward the necrotic core. Though this is conventionally thought of as a limitation of spheroids, recent work has harnessed this property to develop “infarct spheroids” that are exposed to ambient 10% oxygen to generate spatial hypoxia gradients (0–10% oxygen) that mimic the infarct, border, and remote zones after infarction (Figure 2A). These infarct spheroids contained cardiac myocytes, endothelial cells, and stromal cells and were treated with noradrenaline to mimic neurohormonal stimulation after infarction. Infarct spheroids demonstrated similar global gene-expression profiles to human ischemic cardiomyopathy and animal myocardial infarction samples. Furthermore, when compared with control spheroids in ambient oxygen, infarct spheroids exhibited a metabolic shift toward glycolysis, increased stiffness, increased expression of myofibroblast markers, decreased cardiac myocyte contraction amplitude (Figure 2A), and asynchronization of contractions (78).

### Engineered Models of Ischemia-Reperfusion

*In vitro* models have also been engineered to replicate dynamic changes in oxygen characteristic of ischemia-reperfusion. Microfluidic devices are particularly suitable for this application because they contain chambers of small volumes that can be rapidly filled with hypoxic gas or cell medium. For example, a microfluidic device with integrated bioelectronics was used to measure intracellular action potential and extracellular beat rate and propagation velocity in cardiac myocytes cultured in a microchannel. The microchannel was rapidly filled with hypoxic cell medium followed by recovery medium to mimic ischemia-reperfusion. Hypoxic cardiac myocytes demonstrated substantially reduced depolarization times and beat rates, as well as irregular propagation patterns, which recovered within 30 min after reintroducing normoxic cell media (80). A similar microfluidic device was fabricated to contain small chamber volumes that can be quickly filled with hypoxic gas. Gas from the upper chamber diffused across a thin, gas-permeable membrane to reach cells cultured in lower microfluidic channels. This device demonstrated that hypoxic conditions below 5% oxygen induce changes in cardiac myocyte calcium transients, including a decrease in amplitude that could be mimicked using L-type calcium channel antagonists. After a subsequent 10 min of reperfusion with normoxic gas, cardiac myocytes recovered with normal calcium transients (81). Together, these studies suggest that hypoxia induces reversible alterations in cardiac myocyte electrophysiology.

In another model of ischemia-reperfusion, cardiac myocytes were cultured as an aligned tissue on silicone cantilever substrates with embedded strain sensors (Figure 2B). The cardiac myocytes were aligned using microcontact printing, which involves the preparation of silicone “stamps” that are used to transfer extracellular matrix proteins to a substrate in desired geometries. With a spatial resolution of approximately 1 μm, microcontact printing can direct tissue orientation (82–84), single-cell shape (85), and even subcellular structures (86). When the aligned cardiac tissues contracted on the cantilever substrates, the cantilevers deflected, leading to a resistance change in the embedded strain sensors proportional to the contractile stress (87, 88). This system was used to provide real-time measurements of contractile stress in a simulated ischemia-reperfusion injury by switching cells from ischemic media at 1% oxygen to standard media at ambient 21% oxygen. Integrated sensor readouts demonstrated that cardiac tissues stopped contracting during ischemia and displayed a minor recovery of twitch stress during reperfusion. In contrast, pre-treatment with endothelial cell-derived extracellular vesicles was cardioprotective and enabled cardiac tissues to continue contracting during simulated ischemia and exhibit higher recovery of twitch stress after reperfusion (Figure 2B) (79).

In summary, several technologies, including microfluidic devices, hydrogels, spheroids, and strain sensors, have been implemented to mimic the spatial and temporal oxygen gradients characteristic of post-infarct myocardium and subsequently quantify changes in cardiac cell phenotypes. Unlike conventional systems, these approaches can be used to explore oxygen-dependent regional and temporal changes in cellular phenotypes at high resolution and uncover cross-talk between cells in distinct oxygen environments to identify new mechanisms of infarct remodeling.

## *In vitro* Models of Myocardial Fibrosis and Stiffness

Healthy myocardium is a moderately stiff tissue, with an elastic modulus of around 10 kPa. After myocardial infarction, local elastic modulus in the infarcted region increases to 20–100 kPa due to scar formation and fibrosis (89–92). Rat models with coronary artery ligation demonstrate myocardial stiffening over time, with elastic modulus increasing from 18 to 55 kPa (90), and increased stiffness has been observed in the infarct zone as early as 1 day post-infarction (93). In addition to temporal changes, regional stiffness varies between the infarct zone, border zone, and remote zone by 3 days post-infarction (94). Elastic modulus progressively decreases in the border zone at a rate of 8.5 kPa/mm toward remote tissue (90). Because investigating the effects of tissue stiffness with *in vivo* models is confounded by many other concurrent changes, including matrix composition (93), *in vitro* models have been implemented to elucidate the effects of stiffness and other aspects of fibrosis on cardiac cell phenotypes. In this section, we will describe 2-D and 3-D *in vitro* models of cardiac fibrosis that focus primarily on recapitulating uniform or spatiotemporal changes in stiffness.

### 2-D Models With Uniform Stiffness

Because standard polystyrene dishes used for cell culture are nearly five orders of magnitude more stiff than the native myocardium (95), mechanically tunable biomaterials have been developed to mimic the rigidity of healthy or fibrotic myocardium (96). For example, hydrogels are cross-linked, hydrophilic polymers with high water content that are commonly used as cell culture substrates because they can be tuned to resemble the elasticity of soft tissue and allow for efficient mass transfer. Hydrogels can incorporate natural polymer chains, such as mammalian matrix proteins, or synthetic polymer chains, such as polyacrylamide or polyethylene glycol (97, 98). Other biomaterials commonly used to recapitulate physiological or pathological stiffness *in vitro* include elastomers like polydimethylsiloxane (PDMS), which is biocompatible, mechanically tunable, and transparent (99–102).

When cultured on rigid hydrogel or elastomer substrates, cardiac myocytes exhibit disorganized sarcomeres, reduced sarcoplasmic calcium stores (103), lower amplitude calcium currents (103, 104), decreased cell shortening during contraction (103), and a progressive decrease in beat frequency over time (105) when compared to substrates that mimic the elasticity of healthy myocardium. Using traction force microscopy, in which fluorescent beads embedded in substrates are displaced during cell contraction, several studies have established non-monotonic relationships between force generation and substrate rigidity. In these studies, cardiac myocytes generally generate maximum forces on physiological stiffness, which decrease on substrates that are either more soft or stiff in both isotropic (103, 106) and aligned microtissues (107). However, some studies have reported linearly increasing force generation with increased substrate stiffness (101, 108, 109), which may be attributed to differences in experimental methods, such as cell source, biomaterial substrate, or analysis techniques. Similar results are observed in cocultures of cardiac myocytes and fibroblasts on polyacrylamide substrates, in which increased stiffness results in reduced troponin I staining, increased fibroblast density, and poor electrical excitability (106).

Micropatterning has also been used in combination with tunable hydrogel or elastomer substrates to modulate both cellular architecture and substrate stiffness because both of these features remodel concurrently in post-infarct myocardium. Substrate stiffness and cellular architecture has been shown to modulate metabolic activity (99, 100, 107) and mitochondrial structure in cardiac myocytes (102). Microcontact printed hydrogels have also been used to characterize the contractility of single (85) or coupled (110) cardiac myocytes as a function of both cellular architecture and substrate stiffness. At the single cell level, cardiac myocytes with low cell aspect ratios that mimic concentric hypertrophy do more work on stiff substrates that resemble fibrotic myocardium, demonstrating a functional advantage of cell shape remodeling in response to mechanical overload (85). In coupled myocytes, stiff substrates caused increased focal adhesion formation at the cell-cell interface, possibly contributing to cellular uncoupling in post-infarct myocardium (110).

To model both the cellular and biomechanical aspects of fibrosis, tissues have also been engineered with cardiac myocytes and fibroblasts on substrates with tunable stiffness. For example, microcontact printing has been implemented to engineer aligned microtissues on polyacrylamide hydrogels with both cardiac myocytes and fibroblasts. Microtissues generated less work on rigid substrates, irrespective of cell adhesion ligand or presence of fibroblasts, revealing the dominant role of substrate elasticity in regulating contractile output (111). To engineer an artificial infarct boundary, cardiac myocytes and fibroblasts have been cocultured on separate halves of cell culture substrates with rigidities that range from healthy, 1-week post-infarct, and 2- to 6-weeks post-infarct myocardium. The presence of cardiac fibroblasts in this coculture setting attenuated mechanical signal propagation across the infarct boundary in a stiffness-dependent manner (112).

In addition to affecting cardiac myocytes, rigid substrates that mimic fibrotic myocardium also promote fibroblast activation to myofibroblasts. On stiff substrates, cardiac fibroblasts notably activate into a myofibroblast phenotype, exhibiting increased α-SMA coverage (113–116), increased contractile force generation measured through traction force microscopy (117), and increased nuclear localization of the mechanosensitive transcription factors yes-associated protein (YAP) and transcriptional co-activator with PDZ-binding motif (TAZ) (114). Knockdown of YAP and TAZ reversed or attenuated stiffness-dependent changes in cell morphology and function, suggesting YAP and TAZ coordinate fibroblast mechanoactivation (114). In other work, limiting focal adhesion size through microcontact printing was also sufficient to interrupt the recruitment of α-SMA to stress fibers on stiff substrates, indicating that focal adhesion size may control α-SMA localization (113). Studies that establish mechanisms behind fibroblast mechanoactivation may reveal new targets for anti-fibrotic strategies to mitigate adverse remodeling following myocardial infarction.

*In vitro* models have also identified stiffness-dependent secretion of paracrine factors, which may regulate several processes involved in infarct remodeling. For example, cardiac myocytes on stiff substrates secrete more VEGF. Consistent with this finding, media conditioned by myocytes on stiff substrates promotes angiogenesis, including increased migration capacity and tube length of microvascular endothelial cells (118). Fibroblasts cultured on stiff hydrogels and treated with TGF-β have also been shown to upregulate several cytokines, including osteopontin, a known regulator of collagen cross-linking via lysyl oxidase, and insulin-like growth factor 1, which regulates cardiac myocyte hypertrophy. As a result, conditioned media from TGF-β-treated fibroblasts cultured on stiff hydrogels has been shown to induce cardiac myocyte hypertrophy, indicated by increased cell volume, when compared with medium from cardiac fibroblasts without TGF-β, regardless of substrate stiffness (119). This echoes previous work that demonstrates TGF-β may be a more potent regulator of the myofibroblast phenotype than substrate rigidity (116). Together, 2-D models that resemble the elasticity of fibrotic myocardium recapitulate many cellular and molecular events following myocardial infarction.

### 2-D Models With Spatiotemporal Control of Stiffness

2-D models with uniform stiffness do not encompass regional changes in stiffness between the infarct, border, or remote zones following myocardial infarction. Spatial stiffness gradients have been fabricated through graded material cross-linking (120), including gradient-patterned (121–124) or sliding (125, 126) photomasks, layering of hydrogels of different elasticities (127), applying a temperature gradient to PDMS during curing (128), or microfluidic-mixing of prepolymer solutions with different cross-linker concentrations (129). However, most of these have not been implemented with cardiac cell types to model post-infarct myocardium. In one example, a polyethylene glycol hydrogel was patterned with soft and stiff concentric circles using a photomask. Cardiac fibroblasts cultured on stiff regions of the substrate expressed increased α-SMA and collagen when compared with soft regions. Live imaging demonstrated a directional cellular migration toward the inner stiff region. Treatment with a ROCK inhibitor reduced the population of myofibroblasts, demonstrating that the model can be used as an antifibrotic drug screening platform (130). To investigate the effects of pathological matrix stiffening in lung fibroblasts, a stiffness gradient was made from polyacrylamide gels polymerized through gradient photomasks. Human lung fibroblasts cultured on the stiffness gradient show a progressive increase in fibroblast activation, indicated by increased proliferation and matrix synthesis, toward the stiff end of the gradient. Addition of prostaglandin E2, an inhibitor of fibrogenesis, inhibited fibroblast activation (131). Similar phenotypes may also be observed in cardiac fibroblasts over stiffness gradients, though this has not yet been tested.

**Figure 3:**
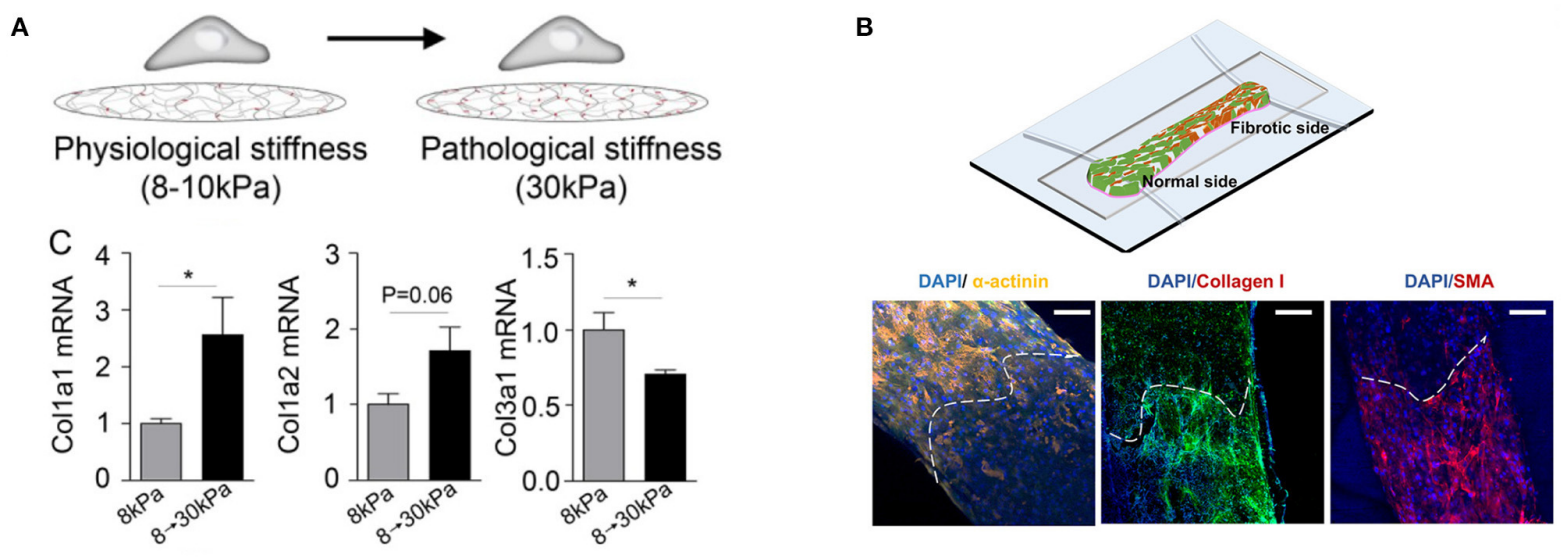
Engineered *in vitro* fibrosis models that modulate stiffness in **(A)** time and **(B)** space. **(A)** A hyaluronic acid hydrogel is polymerized *in situ* upon exposure to UV light to mimic scar maturation after myocardial infarction. Stiffening from 8 to 30 kPa results in collagen turnover (*p < 0.05). Republished with permission of the American Society for Cell Biology, from Herum et al. (115); permission conveyed through Copyright Clearance Center, Inc. **(B)** A heteropolar biowire integrates normal (7.6 kPa) and fibrotic (61.1 kPa) cardiac tissue. The white dashed line indicates the interface between normal and fibrotic regions. Normal regions exhibit increased α-actinin, a component of sarcomeres in cardiac myocytes, while fibrotic regions have increased collagen and α-smooth muscle actin, indicating increased presence of myofibroblasts. Images reproduced with permission of the American Chemical Society, from https://pubs.acs.org/doi/full/10.1021/acscentsci.9b00052 (136). Further permissions related to the material excerpted should be directed to the American Chemical Society.

Mechanical properties can also be controlled *in situ* to model changes in stiffness over time, which is characteristic of infarct scar maturation. This can be achieved with materials that polymerize in response to light exposure (132, 133) or by varying the molecular weight of the cross-linking agent in real-time (134). Engineered models to capture dynamic stiffening have been used to model development, wound healing, and disease (135), but few have been implemented in the context of myocardial infarction. In one study, hyaluronic acid hydrogels seeded with cardiac fibroblasts were modified to dynamically increase in stiffness in response to UV exposure. Dynamic stiffening to model scar maturation resulted in increased cell spreading, α-SMA formation, and collagen I expression (Figure 3A) (115). Although fibroblast activation correlates with increased stiffness in 2-D spatiotemporal models, cardiac myocyte phenotype and function has been relatively unexplored in these settings.

### 3-D Models With Uniform Stiffness

Modulating stiffness in 2-D only exposes one side of cells to the fibrotic microenvironments experienced *in vivo*. To more closely mimic cell-cell and cell-matrix interactions that occur in native myocardium, cardiac cells have been mixed into hydrogels to form 3-D tissues. Similar to findings in 2-D, cardiac myocytes encapsulated in rigid polyethylene glycol hydrogels demonstrate reduced cell shortening and increased relaxation time when compared with soft hydrogels, which was also accompanied by increased intracellular localization of the mechanosensitive transcription factor YAP (133). In 3-D, matrix stiffness promotes fibroblast differentiation into myofibroblasts, demonstrated by increased stellate morphology, α-SMA and collagen type III levels, and gel compaction (137), consistent with findings in 2-D. The simple aggregation of cardiac fibroblasts in 3-D using low-attachment plates has also been shown to induce gene expression changes associated with adverse cardiac remodeling and the extracellular matrix. Conditioned media from 3-D fibroblast aggregates also causes cardiac myocyte hypertrophy relative to media from fibroblasts cultured in 2-D (138), indicating that phenotypes in 2-D do not always translate to 3-D.

Using microfabricated templates, 3-D cardiac tissues have also been engineered with control over cell composition, matrix stiffness, and tissue architecture. In one model, cardiac myocytes and fibroblasts were embedded in collagen hydrogels of varying fibroblast cell densities or collagen concentrations and suspended between uniaxial PDMS microposts. Microposts serve as tissue constraints that promote alignment. Increasing fibroblast density decreased tissue contraction force and hampered beating frequency, as measured by displacement of the microposts (139). In a similar paper, the system was modified to contain biaxial PDMS microposts to generate isotropic cardiac matrices, designed to mimic “diseased” architecture. 3-D microtissues of cardiac myocytes and fibroblasts in isotropic matrices display more stellate morphology, characteristic of myofibroblasts, and more heterogeneous force distribution when compared with “healthy” aligned matrices. Furthermore, increasing the proportion of fibroblasts in the tissues reduces the overall tissue beating frequency, suggesting that both matrix organization and cellular composition regulate cardiac function (140).

Although hydrogels are mechanically tunable, they fail to recapitulate the fibrous architecture of native cardiac extracellular matrix. A 3-D fibrous network functionalized with fibronectin, which anchors cardiac cells *in vivo*, was fabricated through electrospinning. Spin speed was adjusted to tune fiber alignment while photo-initiated cross-linking was used to tune fiber stiffness to mimic physiologic (9–14 kPa) or pathophysiologic (>20 kPa) tissues. Cardiac myocytes in stiff, fibrous networks exhibit slower calcium flux, indicated by increased decay time and increased peak-to-peak irregularity (141). In another example, fibrous scaffolds with varying fiber stiffness were fabricated through two-photon polymerization and seeded with cardiac myocytes that lack expression of cardiac myosin binding protein C, which is thought to play a role in sarcomere sliding during contraction. Mutations in this protein are also associated with hypertrophic and dilated cardiomyopathy. While control cells were able to adapt to the increased load with increasing contraction force, cells with the mutation displayed impaired contraction on stiffer fibers. This work demonstrates the combined effects of mechanical stress and genetic factors on contraction deficits (142).

Interestingly, fibroblasts in 3-D fibrous matrices depart from the conventional relationships established between stiffness and fibroblast activation in 2-D cell culture or 3-D hydrogels (143). In human lung fibroblasts seeded in the same fibrous matrices, increasing fiber stiffness actually reduced proliferation and myofibroblast activation (α-SMA) when compared with cells on soft and deformable fibrous matrices. This is correlated with reduced cell spreading and focal adhesion formation that was also observed with increasing stiffness (144). Fiber density, on the other hand, has been shown to promote differentiation in lung fibroblasts, signified by increased fibronectin synthesis, nuclear localization of YAP, proliferation, and cytokine secretion (145). Similar relationships may also exist for cardiac fibroblasts but have yet to be investigated.

### 3-D Models With Spatiotemporal Control of Stiffness

Engineered 3-D cardiac tissues have also been fabricated with increasing spatiotemporal control over stiffness. A 3-D fibrosis model was developed using the biowire platform, in which cardiac cells are encapsulated in a fibrin-based hydrogel and suspended between a pair of polymer wires that function to promote microtissue alignment. Tissue contractile stress is measured based on the deflection of the intrinsically fluorescent polymer wires. To model healthy (7.6 kPa) or fibrotic (61.1 kPa) myocardium, cardiac myocytes were cocultured with 25 or 75% cardiac fibroblasts, respectively. Fibrotic tissues underwent more rapid compaction and had higher collagen content, disrupted myofibril structures, altered Cx43 distribution, prolonged time to peak, and lower contractile force generation when compared with healthy tissues. To next create a spatially heterogeneous stiffness model, which can mimic the interface between the infarct zone and viable tissue, fibrotic and healthy tissue were integrated at opposing sides of a single biowire platform (Figure 3B). The fibrotic side of the microtissue underwent more rapid compaction, contained increased collagen content and myofibroblast activation, and had slower calcium transients with a lower amplitude compared to the healthy side. In addition, propagation velocity at the healthy side was diminished when compared with uniform healthy biowire tissues. Arrhythmic waves were also observed, especially in the interface region. This platform was also used to screen antifibrotic drugs (136), demonstrating the potential impact of these approaches in drug development.

To alter substrate rigidity over time, one approach is to encapsulate cells into hydrogels that either degrade or cross-link in response to specific wavelengths of light. In one example, a 3-D photodegradable hydrogel was used to demonstrate that valvular myofibroblasts can be redirected into a quiescent phenotype by decreasing stiffness. This work demonstrates fibroblast phenotypic plasticity and the potential role of the mechanical environment in de-differentiating fibroblasts, which has therapeutic applications in resolving fibrotic disease (146). Lastly, one study demonstrated that cardiac myocytes encapsulated in photopolymerizable polyethylene glycol hydrogels do not exhibit differences in cell viability after UV exposure (133), though the impact of progressive stiffening in 3-D on cardiac cell phenotypes has not been further established.

## *In vitro* Models of Pathological Strain

Cardiac cells are constantly under cyclic stretch in the healthy, beating heart. Myocardial infarction results in an initial loss of contractility in the infarct zone followed by arrhythmogenesis, which alters strain rates experienced by surviving cardiac cells, as quantified through echocardiographic imaging (147). To stretch myocytes and non-myocytes *in vitro*, experimental platforms include microchips with stretchable silicone membranes, custom-built bioreactors, or commercially available cell straining units in which strain can be applied to cell culture plates with integrated loading posts.

**Figure 4:**
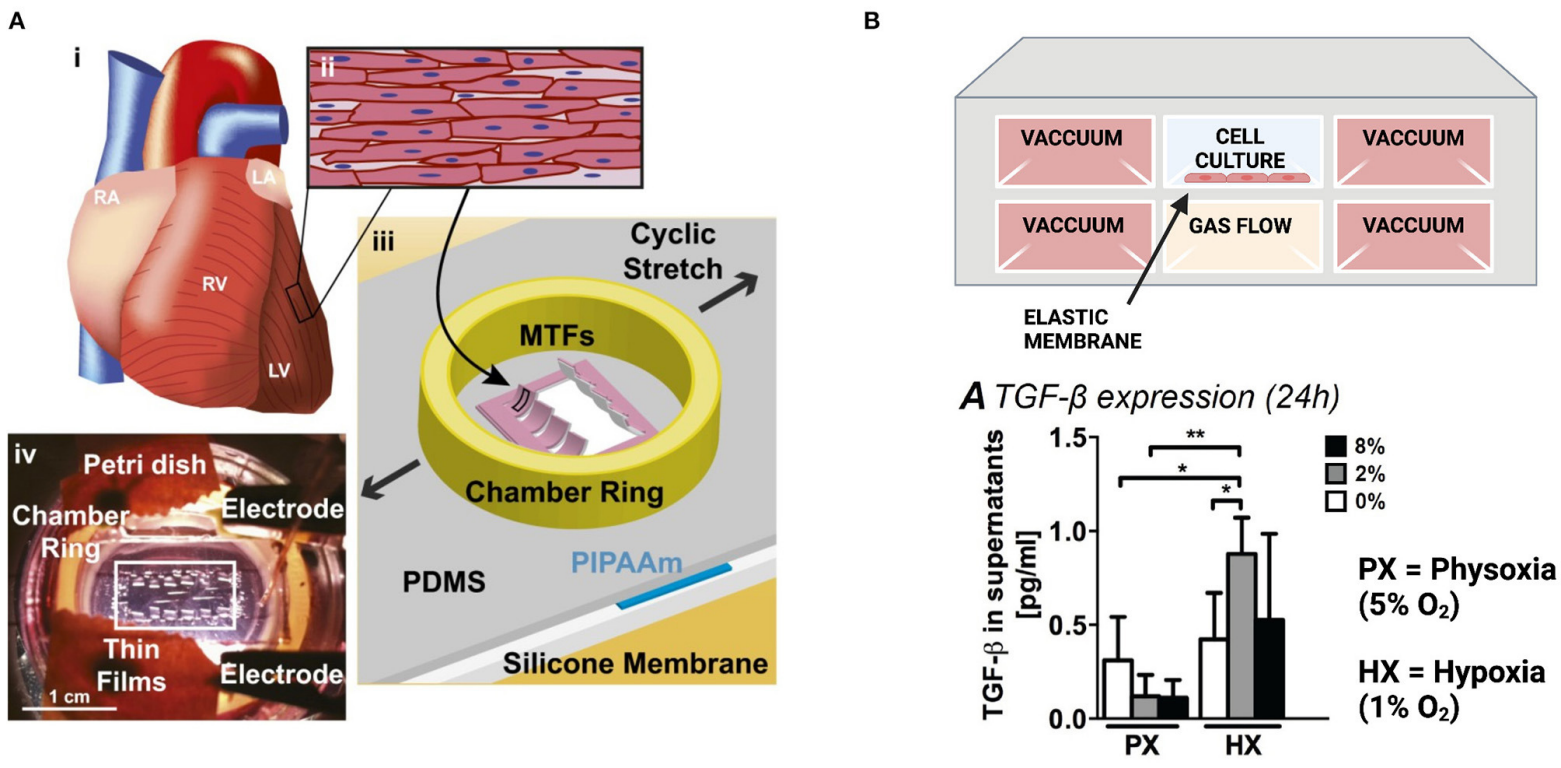
Engineered *in vitro* models to mimic pathological strain post-infarction. **(A)** Muscular thin films on a stretchable silicone membrane provide real-time measurements of contractile stress during stretch. Images reproduced from McCain et al. (154). **(B)** A microfluidic device to evaluate the combined effects of hypoxia and strain on cardiac fibroblasts. Created with BioRender.com. Both hypoxia (1% O_2_) and reduced contractility (2% strain) are required for cardiac fibroblasts to upregulate TGF-β and IL-1β (*p < 0.05, **p < 0.01). Image reproduced from Ugolini et al. (155) (https://creativecommons.org/licenses/by/4.0/).

Chronic cyclic stretch over several days to mimic the diastolic and systolic movement of cardiac muscle has been shown to promote the maturation of “engineered heart tissues,” which are generally defined as cardiac myocytes embedded in hydrogels and cast around uniaxial tissue constraints or circular molds. Stretched heart tissues exhibit increased cell alignment (148–151), sarcomere organization (151, 152), Cx43 expression (150, 151), and contractile force generation (148, 150, 153). In some studies, morphological changes were also observed that indicate cardiac myocyte hypertrophy through increased cell size (148, 152) and mitochondrial density (148). In 2-D aligned cardiac tissues fabricated through microcontact printing, chronic cyclic stretch has also been shown to induce pathological changes in cell aspect ratio and sarcomere alignment, promote gene expression profiles associated with pathological remodeling, and diminish calcium transients and force generation (Figure 4A) (154). Thus, chronic cyclic stretch can be beneficial or detrimental to cardiac myocytes, depending on the specific parameters.

Cardiac fibroblast responses to strain have also been relatively inconsistent. In some cases, stretching activates many hallmarks of cardiac fibrosis, including increased fibroblast proliferation, hydrogel stiffening (156), increased gel compaction and strength (157, 158), extracellular matrix deposition (156–158), and enhanced secretion of TNF-α (159). However, responses are dependent on baseline levels of fibroblast activation, which is highly sensitive to culture conditions. Cardiac fibroblasts that are cultured for 1 day on rigid substrates and have lower initial levels of α-SMA respond to static tensile forces with increased α-SMA, while cells cultured for 3 days with higher basal levels of α-SMA respond to the same force with decreased α-SMA production (160). Consistent with this, fibroblasts grown on soft hydrogels with minimal basal α-SMA expression and exposed to static stretch show elevated α-SMA mRNA levels and expression of various extracellular matrix proteins, including collagen and fibronectin (115). Fibroblasts also show differing proliferative behavior in response to mechanical strain, which may be dependent on baseline α-SMA levels, strain rate (161), ECM composition (162, 163), serum concentration (164), or substrate stiffness (115), which highlights a need for more standardized cell culture methods (165).

To assess the combined effects of strain and hypoxia, cardiac fibroblasts have been cultured in a microfluidic device containing a stretchable, gas-permeable membrane situated above a microchannel for gas flow and between lateral actuation channels (Figure 4B). Uniform hypoxia (1% oxygen) or reduced contractility to mimic post-infarct myocardium (2% strain) are alone sufficient to induce proliferation and collagen type 1 production, although the combined effects of hypoxia and reduced strain are required to trigger fibroblast secretion of IL-1β or TGF-β (155).

Paracrine signals secreted by stretched cardiac cells may also regulate critical aspects of infarct healing. Recent work has characterized the transcriptomic profile of stretched cardiac myocytes, which show differentially expressed genes and regulatory networks that may lead to hypertrophic growth of cardiac myocytes (166). Consistent with this, stretched cardiac myocytes upregulate miR208, a mediator of cardiac hypertrophy, hypertrophic proteins, such as β-myosin heavy chain, and secretion of TGF-β (167). Neonatal rat cardiac myocytes on stretched silicone membranes have also been reported to undergo apoptosis, accompanied by mitochondrial dysfunction (168). One study explored factors secreted by stretched cardiac myocytes by fabricating a coculture device that enables paracrine signaling between cardiac myocytes and fibroblasts while exposing cardiac myocytes to strains that mimic the border zone after infarction *in vivo*. In this device, coculture with stretched cardiac myocytes increases cardiac fibroblast proliferation. A media screen indicated the presence of cytokines such as colony stimulating factor 1 and platelet derived growth factor B, which were sufficient to increase proliferation in fibroblast monocultures (115).

## Outlook

As described above, post-infarct myocardium is characterized by distinct biochemical and biomechanical changes in the cellular microenvironment that vary in both space and time and are thought to contribute to excessive fibrosis, hypertrophy, and arrhythmias. Unlike conventional *in vitro* and *in vivo* models, Organs on Chips are able to dissect the impact of these complex and dynamic changes to the post-infarct microenvironment by offering a unique combination of multi-modal microenvironmental control and accessibility to physiological readouts. For example, the gradient systems described above showed that cardiac fibroblasts migrate toward both ischemic cardiac myocytes (27) and stiffer environments (130), two hallmark features of post-infarct myocardium. These studies also demonstrated that myofibroblast phenotypes can be reduced by inhibiting TGF-β (27) or ROCK (130), suggesting that these molecules or pathways could be exploited as anti-fibrotic therapies. As another example, microfluidic devices that offer precise control over oxygen concentration showed that the electrophysiology of cardiac myocytes becomes irregular in response to hypoxia but can recover after 10–30 min of reperfusion (80, 81). Another Organ on a Chip system showed that pre-treatment with endothelial cell-derived vesicles reduces ischemia-reperfusion injury in engineered cardiac tissues (79). Collectively, these and the other examples highlighted above demonstrate how Organ on a Chip models of post-infarct myocardium are powerful for determining how disease evolution is regulated by spatiotemporal heterogeneity while also serving as platforms for therapeutic development.

Despite the advantages of Organs on Chips, there are still many challenges that limit their widescale adoption for disease modeling and drug discovery. First, some findings in response to hypoxia, stiffness, or strain have produced conflicting results. For example, studies have reported increased (45, 155), decreased (46), or unchanged (44) proliferation of cardiac fibroblasts in response to hypoxia. Similarly, fibroblast proliferation has been shown to increase (155, 161) or decrease (162) in response to strain. This may be due to the inherent heterogeneity of the biological responses or may highlight a need for more standardized experimental methods. There is also a need for more characterization of injured myocardium *in vivo* and *ex vivo* through techniques such as atomic force microscopy, fluorescent oxygen probes, and high-resolution imaging to ensure that Organs on Chips are accurately modeling relevant features of post-infarct myocardium and to establish more universal design parameters.

Another limitation of existing Organ on a Chip models of post-infarct myocardium is their over-simplified architecture. As described above, current *in vitro* models have been predominantly 2-D monocultures that can be micropatterned to control tissue architecture or 3-D cocultured tissues or spheroids with relatively random tissue architecture. Thus, future work should focus on engineering cardiac tissues with distinct control over the positioning of multiple cell types and matrix components, leading to more granular models of post-infarct myocardium. Emerging methods to pattern multiple cell types in 2-D (169) and 3-D (170, 171) can improve the architectural relevance and reproducibility of engineered tissues. 3-D bioprinting has also advanced considerably in recent years to provide increasing structural complexity (172, 173), including spatial gradients in porosity (174) and material and cell composition (175), and can be implemented to make more precise tissue models. Model systems should also strive to incorporate relevant cell types beyond cardiac myocytes and fibroblasts, such as neurons (176) and immune cells (177). In addition, there are other types of spatial and temporal gradients beyond those described above, such as cytokine and chemokine gradients that orchestrate the inflammatory cascade after myocardial infarction. Recent approaches to control gradients of soluble factors using microfluidics (178, 179) or 3-D hydrogels (180) will enable more complex modeling of small molecule gradients in the context of myocardial infarction.

Another limitation of many of the studies described above is their reliance on primary cardiac cells from other species (usually neonatal rats), which have historically been the most accessible cardiac cell source. Cells from neonatal rats exhibit species-specific differences and are relatively resistant to hypoxia, a key feature of the infarct microenvironment (3). Thus, model systems will be improved as the field continues to adopt human induced pluripotent stem cell (hiPSC)-derived cardiac myocytes. In addition to providing human relevance, hiPSC-derived cardiac myocytes can also help identify genetic contributions to post-infarct remodeling (181) and contribute to patient-specific models and treatment regimens. However, a major concern is that these cells demonstrate fetal-like maturity, which is especially problematic for modeling myocardial infarctions, a condition that affects almost exclusively adults. Recent approaches to mature hiPSC-derived cardiac myocytes with electromechanical or biochemical stimuli may help mitigate this concern (182, 183), but achieving adult-like maturity in hiPSC-derived cardiac myocytes remains a major hurdle for the field.

Lastly, Organ on a Chip systems need to be more high-throughput and scalable to be integrated into the drug discovery pipeline. Thus, the field also needs more scalable fabrication methods, such as rapid, multimaterial bioprinting of cardiac biowire scaffolds into 96-well plate formats (184) or the development of substrates with integrated electrodes to streamline electrical stimulation (185). Throughput can also be improved by integrating sensors for real-time readouts of parameters such as tissue contractility (87, 88), action potentials (186), the consumption or secretion of biomolecules (187, 188), or physical aspects of the microenvironment, such as oxygen concentration and temperature (189). These types of multi-sensor systems will provide more continuous and detailed insight into cellular phenotypes in response to drug treatments while also requiring less manual handling, thereby increasing throughput and reproducibility.

In summary, engineered Organ on a Chip models of post-infarct myocardium have exciting potential to address many of the gaps presented by oversimplified 2-D cell culture models and animal models that lack human relevance. As technologies continue to develop, next-generation multi-dimensional models could provide simultaneous control over spatial and temporal changes in the physical, biochemical, and mechanical microenvironment that correspond to the phases of infarct healing. When further advanced with patient-derived cells, scalable fabrication techniques, and integrated sensors, these models have potential to emerge as new standards for disease modeling and drug screening and lead to new breakthrough therapies for mitigating post-infarction remodeling.

## Author Contributions

Both authors were involved in the conceptualization and writing of this manuscript.

## Conflict of Interest

MM is an inventor on US patent US9857356B2 filed by Harvard University and licensed to Emulate, Inc. The remaining author declares that the research was conducted in the absence of any commercial or financial relationships that could be construed as a potential conflict of interest.
